# Double blind, randomized, placebo controlled clinical trial for the treatment of diabetic foot ulcers, using a nitric oxide releasing patch: PATHON

**DOI:** 10.1186/1745-6215-8-26

**Published:** 2007-09-26

**Authors:** Sandra Y Silva, Ligia C Rueda, Gustavo A Márquez, Marcos López, Daniel J Smith, Carlos A Calderón, Juan C Castillo, Jaime Matute, Christian F Rueda-Clausen, Arturo Orduz, Federico A Silva, Piyaporn Kampeerapappun, Mahesh Bhide, Patricio López-Jaramillo

**Affiliations:** 1 VILANO Group. Research Institute, Fundación Cardiovascular de Colombia (FCV), Floridablanca, Santander, Colombia; 2 Department of Chemistry, University of Akron, Akron, Ohio, USA; 3 Fundación Santandereana de Diabetes y Obesidad (FUSANDE), Bucaramanga, Santander, Colombia; 4 Instituto de Seguros Sociales, Bucaramanga, Santander, Colombia; 5 Facultad de Medicina, Universidad de Santander, Bucaramanga, Santander, Colombia

## Abstract

**Background:**

Diabetes Mellitus constitutes one of the most important public health problems due to its high prevalence and enormous social and economic consequences. Diabetic foot ulcers are one of the chronic complications of diabetes mellitus and constitute the most important cause of non-traumatic amputation of inferior limbs. It is estimated that 15% of the diabetic population will develop an ulcer sometime in their lives. Although novel therapies have been proposed, there is no effective treatment for this pathology. Naturally produced nitric oxide participates in the wound healing process by stimulating the synthesis of collagen, triggering the release of chemotactic cytokines, increasing blood vessels permeability, promoting angiogenic activity, stimulating the release of epidermical growth factors, and by interfering with the bacterial mitochondrial respiratory chain. Topically administered nitric oxide has demonstrated to be effective and safe for the treatment of chronic ulcers secondary to cutaneous leishmaniasis. However, due to their unstable nitric oxide release, the topical donors needed to be applied frequently, diminishing the adherence to the treatment. This difficulty has led to the development of a multilayer polymeric transdermal patch produced by electrospinning technique that guarantees a constant nitric oxide release. The main objective of this study is to evaluate the effectiveness and safety of this novel nitric oxide releasing wound dressing for the treatment of diabetic foot ulcers.

**Methods and design:**

A double-blind, placebo-controlled clinical trial, including 100 diabetic patients was designed. At the time of enrollment, a complete medical evaluation and laboratory tests will be performed, and those patients who meet the inclusion criteria randomly assigned to one of two groups. Over the course of 90 days group 1 will receive active patches and group 2 placebo patches. The patients will be seen by the research group at least every two weeks until the healing of the ulcer or the end of the treatment. During each visit the healing process of the ulcer, the patient's health status and the presence of adverse events will be assessed. Should the effectiveness of the patches be demonstrated an alternative treatment would then be available to patients.

**Trial registration:**

NCT00428727.

## Background

Diabetes Mellitus (DM) constitutes one of the most important public health problems with a worldwide impact due to its high prevalence and enormous social and economic consequences. It is believed that there are more than 135 million diabetics, and this number is expected to increase to 300 million in the next 25 years (30% in developed and 70% in developing countries) [1,2].

This epidemic is related to several factors like ethnicity, the longer life expectancy, and the epidemiological and nutritional transition in developing countries due to the urbanization process that brings about obesity and sedentarism [3,4]. In Colombia, the 2002 basic health indicators showed a 2% prevalence of DM. However, it is believed that these numbers are underestimated [5]. Diabetic Foot Ulcers (DFU) are one of the chronic consequences of DM which constitute the most important cause of non-traumatic amputation of the Inferior Limbs (IL), and are associated with high human, social and economic costs [6-11]. It is estimated that approximately 15% of the diabetic population will develop a DFU some time in their life [12-16].

DFU is a consequence of two of the most frequent chronic complications of DM: Peripheral neuropathy and vascular insufficiency [12]. The combination of these factors in association with mechanic extrinsic and intrinsic aggressions, like feet bone deformations, triggers the ulcer formation. This polyfactorial etiopathology explains the multidisciplinary approach required for this disease [17-19].

The early detection of risk factors for DFU may prevent their appearance [20-28]. This is why the monitoring of neuropathic, vascular and muscle-skeletal functions [29], the reinforcement of self care [30], the control of glycemic levels and the prevention of traumatic foot lesions [21,23,24,31], have a positive impact on the prevention of this pathology.

The wound healing process involves the interaction of multiple cellular groups, extra cellular matrix molecules and growth factors [32], and is affected by vascular insufficiency, the severity of the lesion and the presence of infection [18,24].

Immune and wound healing mechanism dysfunctions have been described in diabetic patients [33]. This alteration of the immune response is characterized by a decrease in the adherence of leukocytes to the capillary endothelium, chemotaxis damage and a reduction in the ability of polymorphonuclear cells to phagocyte and destroy bacteria due to a minor production of Nitric Oxide (NO) [34,35]. Diabetic patients also show a diminution in fibroblasts activity provoked by the unavailability of glucose for the aerobic metabolism, leading to inadequate fibrous collagen tissue proliferation [36]. The imbalance in these processes has been described as the main cause for the appearance and persistence of DFU [37].

Alternative therapies for DFU have been proposed, such as tissue engineering [38], growth factors [39], hyperbaric oxygen therapy [40,41] and ketanserine [42,43]. Various types of products have also been used to keep the wound dry and covered (hydrogels, hydrocolloids, alginates and foams) [44], however, there is no effective treatment for this pathology [45,46].

In normal conditions, NO is continuously produced [47] and is tightly linked to many physiological processes, among which wound healing. It has been described that NO stimulates collagen synthesis [48], triggers the release of chemotactic cytokines [49], and has an important microbicidal effect by interfering with the enzymatic processes of the bacterial mitochondrial respiratory chain [50]. It also increases blood vessels permeability [51-54], promotes angiogenic activity, and stimulates the release of epidermical growth factors [37,55].

Various studies, performed in murine models, have demonstrated the role of NO in the healing process. The levels of the final metabolic products from NO (nitrite and nitrate) rise during the first two days more than subsequently in the liquid recovered from the sponges previously placed in the subcutaneous tissue of healthy subjects' wounds [56], increase that is not observed in diabetic subjects [54], suggesting an impairment in the cutaneous production of NO in diabetic individuals. The topical use of NO accelerates the wound healing process of excisional wounds [54,57] while NO inhibitors increase the healing time of these lesions [48,58]. The use of colloid NO donors in diabetic subjects shows an adequate granulation and closing of wounds [59,60].

Our experience in the treatment of cutaneous leishmaniasis with s-nitroso-N-acetilpenicilamine (SNAP), a NO donor, shows a beneficial effect without adverse events [61]. Moreover, a clinical trial is being conducted to study the effectiveness of a controlled NO releasing patch in the treatment of cutaneous leishmaniasis [62]. Currently, there are no studies using topical NO donors in humans with diabetic ulcers. Though the use of NO donors in healthy volunteers has shown an adequate NO diffusion rate to dermis, and an increase in the microcapillar blood flow [63], this method has demonstrated not to be effective due to the short half life and release span of NO [61].

This difficulty in controlling the stability and release of NO has led to the development of a new NO releasing patch (NOP). This device, produced by the electrospinning technique [64], is a multilayer polymeric transdermal patch in which the principal components are stabilized and encapsulated in nanofibers which guarantees a constant NO release upon its hydration.

Since it is well known that the system generates NO almost immediately, nitrite ions are bound to an ionic exchange resin (DOWEX^®^) in order to stabilize them, allowing a constant release of 3.5 μmol of NO during 12 hours or more depending upon the dosage [64]. This device has been tested by our group for the treatment of cutaneous leishmaniasis obtaining encouraging results in the healing process with no report of serious adverse events [61]. Since there is no specific effective treatment for DFU our group proposes to investigate the topical use of NOP, elaborated by the electrospinning technique, for the treatment of DFU.

### Objectives

#### General Objective

To evaluate the effectiveness and safety of NOP for the treatment of DFU.

#### Specific Objectives

1. To evaluate the healing process of DFU using a NOP compared with placebo.

2. To assess the healing process of infected DFU using a NOP compared with placebo.

3. To identify adverse events associated with the application of NOP.

## Methods and design

Double blind, randomized, placebo controlled clinical trial.

### Sample size

The sample was calculated according to the arccosine formula considering a power of 80% and a type I error of 0.05. Assigning a successful rate of 30% in the control group and 60% in the active group, 95 participants will be needed. After adjusting for a loss rate of 5%, the total number of patients that must be recruited is 100 (50 patients per group).

### Population

The population will be composed of patients with a confirmed diagnosis of DM type 1 or 2 who present DFU, meet the inclusion criteria and don't present any criterion that could exclude them.

### Inclusion criteria

1. Men and women 18 years or older.

2. Capacity of attending the visits at the research site.

3. Confirmed diagnosis of DM type 1 or 2 according to the guidelines from the American Diabetes Association (ADA).

4. Presence of 1 or more DFU, less than 15 cm in its biggest diameter, with a Texas University score ≤ 2.

5. Pharmacological treatment for glycemic control.

6. Willingness to participate in the study and to sign the informed consent form.

### Exclusion criteria

1. Unconfirmed DM diagnosis.

2. Any pathology that, based on the judgment of the researcher, could alter the course of DFU (neoplasias, immunological disorders, etc).

3. Renal insufficiency requiring dialysis treatment.

4. DFU with aTexas score >2.

5. Infected DFU with clinical or paraclinical findings suggesting osteomielytis.

6. Critical ischemia of IL diagnosed by Doppler ultrasound and defined by ankle/arm index < 0.5.

7. Clinical findings suggesting complicated venous insufficiency of IL.

8. Distal necrosis of the limb with the ulcer.

9. Pregnant or breastfeeding women.

10. Mentally or neurologically disabled patients that are considered not fit to approve their participation in the study.

11. Refusal to give informed consent.

### Study development

See table 1.

**Table 1:** Schedule of activities

	**Follow-up visits**							
**PROTOCOL ACTIVITIES**	**Screening Visit**	**Initial Visit**	**Visit 1**	**Visit 2**	**Visit 3**	**Visit 4**	**Visit 5**	**Final Visit**
**SCHEDULE OF VISITS**		0	15 ± 5	30 ± 5	45 ± 5	60 ± 5	75 ± 5	90 ± 5
Clinical History	X	X	X	X	X	X	X	X
Physical Examination	X	X	X	X	X	X	X	X
Inclusion/Exclusion criteria	X							
Informed Consent	X							
Clinical assessment of vascular compromise	X	X	X	X	X	X	X	X
Clinical assessment of neuropathic compromise		X						X
Laboratory tests		X						X
Ankle/arm index	X							
Randomization		X						
Treatment administration		X	X	X	X	X	X	
Clinical assessment of the lesion	X	X	X	X	X	X	X	X
Measurement of ulcers	X	X	X	X	X	X	X	X
Photographic registration		X	X	X	X	X	X	X
Education of patients	X	X	X	X	X	X	X	X
Documentation of adverse events	X	X	X	X	X	X	X	X
Recording of concomitant medications	X	X	X	X	X	X	X	X

### Logistic phase

This phase will be managed by the physician in charge of the study and a professional nurse and will include the following activities.

1. Acquisition of the materials required for the development of the project.

2. Elaboration of flyers, promotional and educative material, procedures manual and Case Report Format (CRF).

4. Training of personnel that will participate in the study.

5. Treatment randomization

The treatment randomization will be realized by the epidemiologist of the Fundación Cardiovascular de Colombia (FCV). This randomization will be done in blocks in order to avoid long sequences of patients assigned to the same group and to reduce when possible, some of the bias inherent to the simple randomization process. Additionally, this randomization in blocks will facilitate the execution of interim analyses.

### Recruitment phase

Patients will attend the screening visit at the FCV or at the offices of the physicians involved in the study.

### Screening visit

During this visit a complete medical check-up, based on universally accepted techniques, will evaluate risk factors, DM complications, vascular and neuropathic compromise, medications and other therapies used for the treatment of DFU. The ankle/arm index will be obtained to rule out the presence of critical IL ischemia. The inclusion/exclusion criteria will be applied and the selected candidates informed about the study after which they will sign an informed consent form. A blood sample will be withdrawn to determine the percentage of glycosilated hemoglobin (HbA1c), the levels of creatinine and fasting glucose, the hemogram, and the lipid profile.

The laboratory of the Research Institute of FCV and the laboratories of every other center will be in charge of the processing of blood samples. All the tests will be elaborated using standard techniques.

1. Glycosilated hemoglobin: Chromatography.

2. Creatinine: Spectrophotometry.

3. Hemogram: Manual method.

4. Lipid profile: Spectrophotometry.

5. Fasting glucose: Spectrophotometry.

### Initial visit

The selected patients will be scheduled for the initial visit. In this visit the patients will be randomly assigned to one of the two groups. Group 1 will receive NOP and Group 2 placebo patches. A complete medical evaluation will be performed, the ulcers measured, and their pictures taken. Neuropathy will be assessed using the modified validated neuropathic impairment score (NDS) [65]. Before taking the pictures, a graduated ruler will be placed next to the ulcers with a sticker marked with the identification code of the participant and the date of the visit. The first patch (active or placebo) will be applied on the lesion. All the information obtained will be registered in the CRFs. The treatment previously prescribed for DM or other concomitant pathologies, and the medical, surgical or orthopedic therapies for DFU will be continued with the exception of topical treatment. The researchers will follow the guidelines of the International Diabetic Foot Consensus (CIPD). The patient and his/her family will receive information regarding the treatment of DFU, the correct technique for the daily application of the patches and the identification and notification of adverse events.

### Follow-up visits

The treatment will last 90 days. During this period, the patients will be seen by the research group at least every two weeks until the healing of the ulcer or the end of the treatment. The frequency of the visits will vary depending on the clinical evolution of the patients. During each visit the healing process of the ulcer, the patient's health status and the presence of adverse events will be assessed. The patch will be applied and the evolution of the ulcers photographically registered. The maximum and minimum diameters of the ulcer will be measured using a graduated ruler and then registered in the CRF. The technique for the application of the patches, the identification of adverse events and the importance of reporting them, will be emphasized.

### Final visit

The final visit will take place at the end of the treatment or before in case of complete healing of the ulcer. A thorough medical evaluation, including laboratory tests will be performed along with the assessment of vascular and neuropathic compromise. The presence of adverse events will also be evaluated. The evolution of the ulcers will be documented by measuring their maximum and minimum diameters and by photographing them.

### National Coordination from Monitoring Center

Every six months a researcher from FCV will visit each center to evaluate the fulfillment of the protocol and the accomplishment of the Good Clinical Practice.

### Data base depuration phase

After completing all the data entry to the CRF the results will be audited and the detected errors evaluated and corrected. The information will be entered in two different databases by two different people and the records compared to detect any discrepancy using a statistical software (Epi-Info 6.04). The mistakes will be corrected according to the CRF, and the corrections registered.

Each patient will be identified using an internal code. All the photographs will be processed using a digital edition software.

### Statistical analysis

For the statistical analysis, Stata 8.0 will be used. The descriptive analysis will be composed of medians and proportions according to the nature of the variables, with their respective 95% confidence intervals. As a dispersion measurement the standard deviation will be calculated. The distribution of the variables will be studied using the Shapiro-Wilk test and the homocedasticity of the variances with the Levene test. To detect any difference between the groups, a T-test or a Mann Whitney test will be performed according to the distribution of the variables. The categorical variables will be compared using the Chi^2 ^test or the exact Fisher's test. If required, a model of multiple logistic regressions or a covariance analysis will be realized. Two interim analyses will be performed when 35 and 70% of the total sample is collected to determine differences in effectiveness and safety between the groups.

### Endpoints

At the end of the treatment the following endpoints will be evaluated:

1. Ulcer reduction percentage.

2. Complete cure of the infection that was present before the treatment.

3. Infection of the ulcers during the treatment.

4. Presence of adverse events related to the application of the patches.

### Final report

The results of the study will be evaluated and discussed and a final report presented to the Federación Diabetológica Colombiana (FDC), entity that is sponsoring the project. The results will be submitted for publication and presented in congresses and scientific meetings.

### Ethical aspects

This study will be conducted in accordance with the Declaration of Helsinki and with the Colombian legislation as per the Resolution 8430/93 from the Ministry of Health. Prior to the admission of the patients in the study, the objectives and the methodology will be explained and a written informed consent obtained. The study was approved by the Research Ethic Committee of the FCV (Act# 075/April 27/2004). The patients' right to confidentiality will be maintained in all the phases of the study.

### Evaluation and management of adverse events

During the visits the patients will be asked about adverse events. Each adverse event will be classified by the physician as serious or non-serious. A serious adverse event should meet one or more of the following criteria:

1. Death

2. Life-threatening

3. Hospitalization or prolongation of existing hospitalization

4. Persistent or significant disability/incapacity

The presence of a serious adverse event that puts the patient's life at risk and/or requires immediate medical or surgical procedure will call for the discontinuation of the treatment and the initiation of the pertinent medical management. The investigator will notify the Adverse Event Committee (AEC) of the FCV of any serious adverse event within 24 hours of learning about it.

A non-serious adverse event will be classified as follows:

1. Mild: The patient is aware of his/her symptoms and/or signs, but those are tolerable. Medical intervention or specific treatment is not required.

2. Moderate: The patients present troubles that interfere with his/her daily activities. Medical intervention or specific treatment is required.

3. Severe: The patient is unable to work or to attend his/her daily activities. Medical intervention or specific treatment is required.

The possible relationship between the adverse events and the tested medication will be classified by the investigator on the basis of his/her clinical judgment and the following definitions:

1. Definitely related: Event can be fully explained by the administration of the tested medication.

2. Probably related: Event is most likely to be explained by the administration of the tested medication rather than other medications or by the patient's clinical state.

3. Possibly related: Event may be explained by the administration of the tested medication or other medications or by the patient's clinical state.

4. Not related: Event is most likely to be explained by the patient's clinical state or by the use of other medications, rather than the tested one.

All the events will be reported to the AEC that, depending on its criteria, will decide the continuity of the patient in the study and therefore the breaking of the code. Although the project has been designed to minimize the inherent risks, any adverse event related to the study medication will be carefully evaluated by the AEC and the costs generated by the required treatment covered by the study.

## Competing interests

The author(s) declare that they have no competing interests.

## Authors' contributions

PLJ made substantial contributions to the conception and design of the study, will be responsible for the administration and direction of the project, the analysis and interpretation of data and will give the final approval of the manuscript to be published. DS, who participated in the design of the project, will be responsible for the production and analysis of the NO releasing electrospun nanofiber patches. GAM made substantial contributions to the conception and design of the study and will be responsible for analyzing and interpreting the final data. ML, PK and MB were in charge of the development of the NO releasing patches, the drafting of the manuscript and will participate in the analysis and interpretation of data. JCC, CAC, JM, AO will be responsible for overseeing the logistics of the clinical trial and will interpret the final data. FAS contributed to the conception and design of the study, performed sample size calculation and randomization and will analyze the data obtained from the clinical trial. SYS, CFR and LCR made substantial contributions to the conception and design of the study and were involved in drafting the manuscript. All authors read and approved the final manuscript.
